# Family planning knowledge and practices among physicians in academic centres in Johannesburg, South Africa

**DOI:** 10.4102/jcmsa.v4i1.343

**Published:** 2026-10-15

**Authors:** Alison B. Rothman, Shastra A. Bhoora, Jarrod Zamparini

**Affiliations:** 1 Department of Obstetrics and Gynaecology, Faculty of Health Sciences, University of the Witwatersrand, Johannesburg, South Africa; 2 Department of Obstetrics and Gynaecology, Faculty of Medicine, Baylor College, Texas, United States of America; 3 Department of Internal Medicine, Faculty of Health Sciences, University of the Witwatersrand, Johannesburg, South Africa; 4 Department of Obstetric Internal Medicine, Charlotte Maxeke Academic Hospital, Johannesburg, South Africa

**Keywords:** family planning, contraception knowledge, contraceptive counselling, long acting reversible contraception, physicians, registrar training

## Abstract

**Background:**

Specialists in Internal Medicine may manage women of childbearing age, but data regarding their family planning knowledge and prescribing practices are lacking. This study aimed to assess the family planning knowledge and prescribing practices of Consultants and Trainee Physicians (Registrars) in three academic hospitals in Johannesburg and to identify barriers to effective contraceptive counselling for women with chronic medical conditions.

**Methods:**

A cross-sectional online survey was conducted among Consultants and Registrars in Internal Medicine at the University of the Witwatersrand. Likert-scale items assessed attitudes to contraceptive prescribing, and single-best-answer questions assessed contraceptive knowledge. Descriptive statistics summarised demographics, and Poisson regression identified factors associated with higher scores; *p* < 0.05 was considered statistically significant.

**Results:**

Of the 82 respondents (35% response rate), with a median age of 32 years (interquartile range 30–37), 47 (57.3%) were female, and 54 (65.9%) were Registrars. Fifty respondents (61%) had high attitude scores, but the median knowledge was 53.8%, and only 34/82 (41.5%) respondents achieved the pragmatic threshold of ≥ 10/13 for adequate knowledge. Key barriers were high workload and lack of sexual and reproductive health training. Married respondents were more likely than single respondents to have high attitude scores (PRR 1.83, 95% CI 1.05–3.17), while male doctors had higher practice scores than female doctors (PRR 1.92, 95% CI 1.14–3.26). No variables were associated with high objective assessment scores.

**Conclusion:**

Gaps in contraceptive knowledge and decision-making amongst Consultants and Trainee Physicians highlight the need for improved education to support comprehensive care for women of childbearing age, especially those with medical comorbidities.

**Contribution:**

This study highlights the importance of collaboration between Internal Medicine and Gynaecological specialities in a combined effort to reduce unwanted and high-risk pregnancies to promote the overall health of women, especially those living with medical conditions.

## Introduction

Access to comprehensive contraceptive counselling has personal, social, economic and medical advantages. It empowers women and provides reproductive autonomy, enhancing the economic and social opportunities of women.^1^ The World Health Organization (WHO) has stated that ‘contraceptive use can prevent unplanned pregnancies, reduce the number of abortions and lower the risk of maternal death and disability’.^2^ The prevention of unplanned pregnancies is especially pertinent to South Africa (SA), where 51.6 % of pregnancies in 2019 were unplanned.^3^

The WHO estimates that increasing contraceptive use will ‘reduce the risk of maternal deaths by over 40%’,^4^ a view also supported by a study in The Lancet Global Health, which analysed population-level data from 193 countries between 1950 and 2010.^5^ The Lancet study showed that for every 10% increase in contraceptive use, there was a 36% reduction in maternal mortality.^5^

An issue that is pertinent to this study is the concept of unmet need: when women of childbearing age are sexually active without using any form of contraception, despite not wanting or planning to conceive.^6^ It is estimated that globally, there are 215 million women aged 15–49 years who want to avoid pregnancy but are not using a reliable method of contraception.^7^ The State of the World Population Report (2022) revealed that nearly 50% of all pregnancies are unintended,^8^ underscoring the substantial gap in access to and use of contraceptives. This gap has more severe consequences for women with pre-existing medical conditions, as unplanned pregnancies increase the risk of adverse maternal outcomes.^6^ Furthermore, medical conditions (including non-pregnancy-related infections and hypertension) are the leading cause of maternal death in South Africa, and there has been a sustained increase in deaths because of pre-existing medical conditions.^9^

In South Africa, specialists in Internal Medicine (Physicians) are usually the primary healthcare providers for patients with chronic medical conditions, including women of childbearing age with conditions that may impact pregnancy. The contraceptive prescription practices of these physicians are therefore pertinent and particularly important for women with co-morbid medical conditions. These women face a higher risk of complications during pregnancy^5^ and therefore require comprehensive and well-informed contraceptive counselling and family planning support.

We aimed to establish the family planning knowledge and practices of qualified Physicians (Consultants) and trainees (Registrars) in Internal Medicine, in academic hospitals in Johannesburg. Additionally, we sought to identify barriers that may hinder Physicians in providing adequate contraceptive counselling.

## Research methods and design

### Study design and setting

This was a cross-sectional study using an online survey, conducted among all Consultants and Registrars in the Department of Internal Medicine at the University of the Witwatersrand, Johannesburg (Wits). The clinicians sampled were working in one of the three academic hospitals affiliated with Wits. Clinicians practising in Internal Medicine in these hospitals but not affiliated to Wits (e.g. medical officers) were not included in the study population.

### Data tool and collection

Data were collected using an online questionnaire using Google Forms (Google LLC, California). The survey is available as Online Appendix 1. The survey was distributed to all 234 eligible physicians (124 Consultants and 110 Registrars) via existing departmental email lists and WhatsApp (Meta Platforms, Inc.) groups. The responses were accepted over 6 weeks between 15 February 2024 and 31 March 2024.

All respondents provided informed consent before completing the survey. Survey responses were stored in Google Forms and could only be accessed by the research team.

Using the expected survey response rate of 33% described by Nulty,^10^ we aimed to collect at least 77 responses. A second sample size calculation, using liberal conditions for online survey responses (an 80% confidence interval [CI] and 10% margin of error), determined that 35 responses would be adequate.

The survey consisted of three sections. The first section collected respondent demographic data. The second section explored participants’ self-reported attitudes and practices relating to contraception using a series of Likert-scale questions. These questions assessed whether participants recognised the importance of contraception, whether they included contraception counselling in the assessment and management of patients and what factors they perceived as limiting their confidence in prescribing contraception. The third section consisted of ten multiple-choice questions designed to assess the depth of knowledge of contraception.

The survey was designed by the first author (AR) and reviewed and edited by SB (a specialist Obstetrician & Gynaecologist with experience in contraceptive care) and JZ (a specialist physician with experience in preconception medicine and the management of medical problems in pregnancy). It was externally validated for both content and clarity by three registrars in Obstetrics & Gynaecology, and two specialist physicians in academic practice – one with experience in medical education, based at a different medical school in South Africa, and one based in the United Kingdom with experience in preconception medicine.

### Data analysis

Data were analysed in Stata/BE-basic edition 17.0 (StataCorp, College Station, Texas, United States, and R version 4.2.0). Descriptive statistics, including frequencies and percentages, were used to summarise participant demographics. Categorical variables such as biological sex, relationship status and professional subspeciality were captured, while continuous variables were reported as medians with interquartile ranges (IQR). Data analysis followed these steps:

- Attitudes towards contraceptive prescribing were assessed using seven Likert scale questions, each scored 1 to 7, giving a maximum score of 49. For the regression analysis, a high attitude-domain score was defined as a total score of ≥ 35/49, corresponding to an average item response of at least 5 across the seven items. As some items assessed perceived barriers, including workload and lack of training, the score should be interpreted as a broad measure of responses within the attitude domain rather than as a unidirectional measure of positive attitude.
- Contraceptive prescription practices were measured using a four-item Likert scale, each scored from 1 to 4, giving a maximum score of 16. For the regression analysis, a high practice score was defined as > 8/16, corresponding to scores in the upper half of the practice scale.
- Knowledge of contraceptive use was assessed with 10 single-best-answer and 3 multiple-choice questions. Responses were scored as 1 for correct and 0 for incorrect, giving a maximum score of 13. For the regression analysis, a higher knowledge score was defined as ≥ 10/13, approximating a score of 75%. This threshold was selected pragmatically as a descriptive benchmark for adequate knowledge and was not based on prior validation of the questionnaire.^7^

These dichotomised outcomes were used to identify factors associated with higher scores. Poisson regression with robust error variance was used for each model. Variables with *p* < 0.2 in univariable analyses were included in the multivariable models to avoid premature exclusion of potentially important predictors, with age included a priori for the practice model. In all three regression models, a *p*-value of < 0.05 was considered statistically significant. Internal consistency of each scale was assessed using Cronbach’s *α*.

### Ethical considerations

Ethical clearance to conduct this study was obtained from the University of Witwatersrand and the Human Research Ethics (Medical), HREC-M (ref. no. M230807), as well as the Academic Head of the Department of Internal Medicine at the University of the Witwatersrand, Johannesburg.

## Results

The survey was sent as an online link to all 234 clinicians (124 Consultants and 110 Registrars) in the Department of Internal Medicine at the University of the Witwatersrand, Johannesburg. Eighty-nine responses were received, of which 7 were incomplete (one or more questions were left unanswered), resulting in a final cohort of 82 (*n* = 82) respondents.

The median age of the cohort was 32 years (IQR 30–37) and more women (*n* = 47: 57.3%) than men (*n* = 35: 42.6%) responded to the survey (Table 1). There were more Registrars (*n* = 54, 65.9%) included in the study than Consultants (*n* = 28, 34.1%). Among the Registrars, 20 (37.5%) were in their first year and 10 (18.9%) in their 4th year of training. The median year of qualification was 2018 in the Consultant group, and most respondents were married (*n* = 47, 57.3%).

**TABLE 1:** Description of enrolled doctors.

Characteristic	*n*	%	Median	IQR
**Age (years)**	-	-	32	30–37
**Gender**				
Female	47	57.3	-	-
Male	35	42.6	-	-
**Level of training**				
Registrar	54	65.9	-	-
Consultant	28	34.1	-	-
**Relationship status**				
Single	21	25.6	-	-
In a relationship	10	12.2	-	-
Married	47	57.3	-	-
Divorced	3	3.7	-	-
Not disclosed	1	1.2	-	-
**Year of training† (among registrars, *n* = 54)**				
1st year	21	38.9	-	-
2nd year	12	22.6	-	-
3rd year	10	18.5	-	-
4th year	10	18.5	-	-
5th year	1	1.9	-	-
Year of qualification (among consultants, *n* = 28)	-	-	2018	2011–2020
**Qualified 2020 or later (among consultants, *n* = 28)**				
No	19	67.9	-	-
Yes	9	32.1	-	-

IQR, interquartile range.

† , The registrar programme is 4 years of training, but some may have had their time extended to 5 years.

The median attitude-domain score was 35/49 (IQR 33–38). Using the pre-specified threshold of ≥ 35/49, 50 respondents (61.0%) had a high attitude score. Figure 1 shows the distribution of attitude scores per question. More than 50% of respondents agreed that ‘the workload in my facility is too great to provide contraceptive counselling to my inpatients’. Lack of training in sexual and reproductive health was also identified as a limiting factor. The Cronbach’s alpha for the attitude scale was 0.55.

**FIGURE 1:**
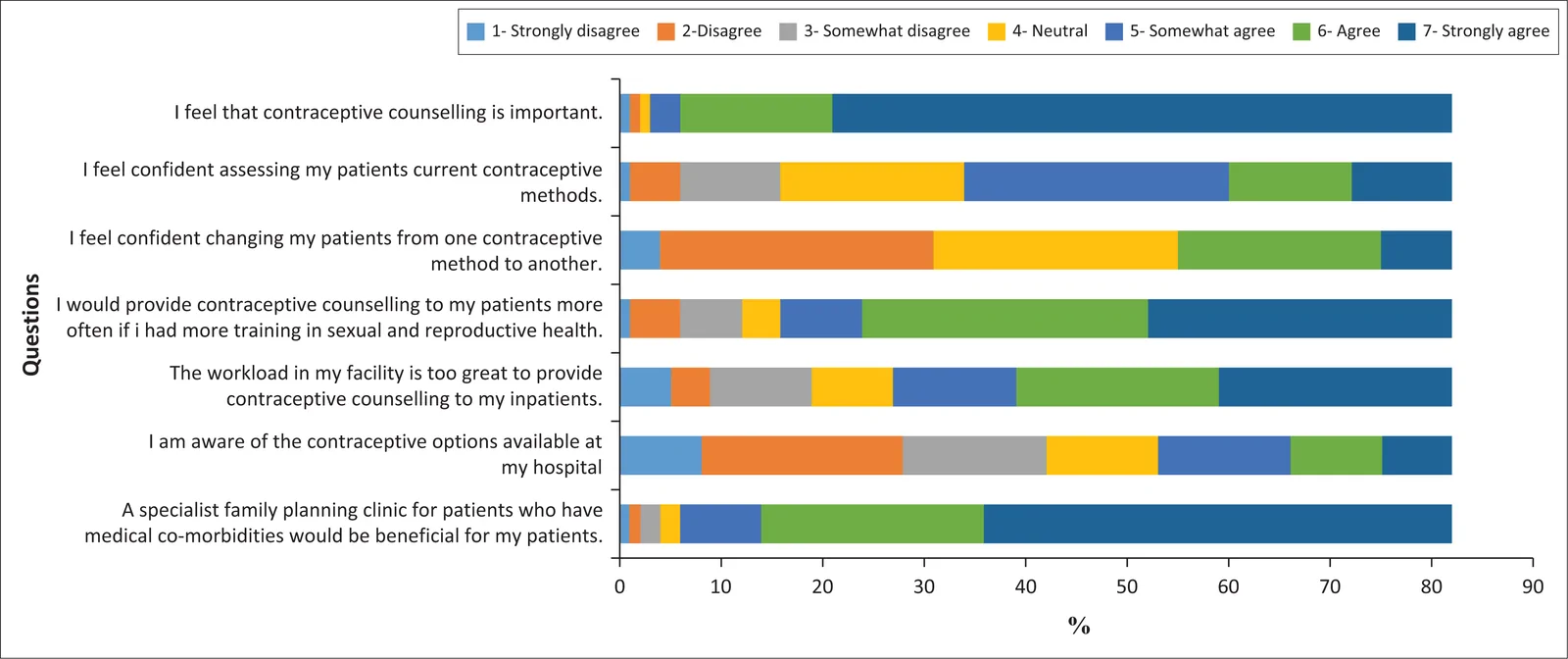
Attitude towards contraception (Cronbach alpha = 0.55).

As shown in Figure 2, only 10% of the respondents said they screen patients for pregnancy at every visit. Recording the first day of the last menstrual period was only reported to be done at every doctor’s visit in 2% of appointments. Doctors who said that they used the World Health Organization Medical Eligibility Criteria (WHO MEC)^8^ every time they see women of reproductive age was also low at 5%. The median total practice score was 8 (IQR 7–9) out of a maximum score of 16. High scores in the subjective section were equivalent to item scores of 3 or 4 across all items in the practice scale, meaning they either said that they will either agree with the statement most or all of the time. Thirty-four (34) respondents (41.5%) had high scores (> 8).

**FIGURE 2:**
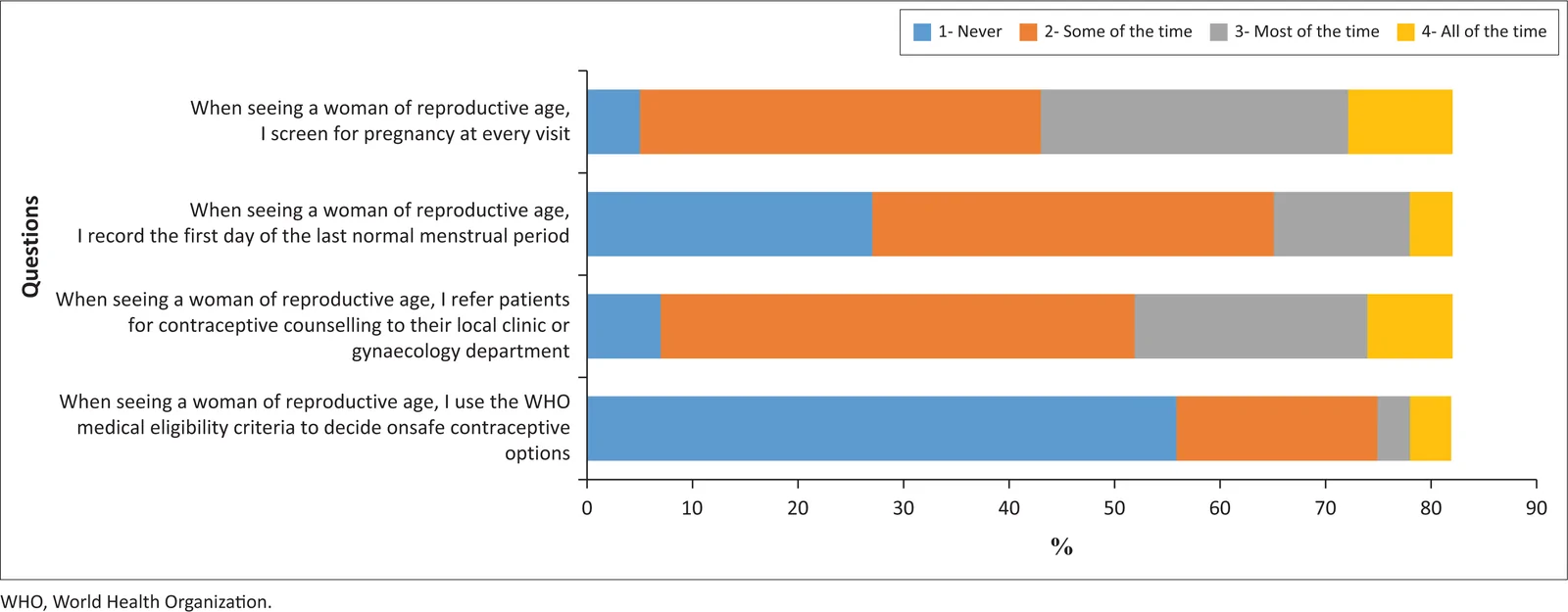
Practice towards contraception (Scale in %).

In a multivariable model, males were associated with high practice scores compared with females (PRR 1.92 [95% CI 1.14–3.26], *p* = 0.015). Among Consultants, there was a non-statistically significant trend towards higher practice scores among those in general medicine compared to those in subspecialities.

The median knowledge score was 7/13 (IQR 6–10), equivalent to 53.8%. Using the pragmatic threshold of ≥ 10/13 for adequate knowledge, 34/82 (41.5%) respondents achieved this level. The question that was most frequently answered incorrectly related to the dose adjustment of antiepileptic drugs when starting combined oral contraceptives. Respondents were able to identify the absolute contraindications of both oral contraceptives and hormonal intrauterine systems. The safest contraceptive options for venous thromboembolism were answered correctly by 33% of respondents. In both univariate and multivariate analyses, none of the variables were associated with high objective assessment scores.

Details regarding the specific questions are available in the questionnaire, which appears in Online Appendix 1.

## Discussion

Our study found that although Physicians acknowledge the importance of contraceptive counselling,^11^ there is an opportunity to enhance their knowledge and improve their prescribing practices. Female respondents prescribed contraception more than their male colleagues. This is likely as a result of them having personal experience with their own reproductive health. When comparing relationship statuses, it was found that married women prescribed contraception more often than their single counterparts. This correlated with the survey of Internal Medicine residents in Los Angeles County in 2009 in the United States that Lohr et al. conducted. They also found that female gender increased the likelihood of contraceptive counselling being offered by the doctors to their patients.^12^ The correlation of these studies proves that female physicians approach patients with the same sex in similar ways in SA and in America. The relatively low internal consistency of the attitude scale should be considered when interpreting these findings. The Cronbach’s alpha of 0.55 suggests that the attitude items may not have measured a single homogeneous construct (Table 2). Rather, they captured related but distinct aspects of contraceptive care, including perceived importance, perceived responsibility, confidence, workload constraints and training barriers. The attitude score should therefore be interpreted as a broad descriptive measure rather than as a psychometrically validated scale of a single attitude construct.

**TABLE 2:** Factors associated with high attitude scores (≥ 35) among registrars and consultants in internal medicine.

Variable	High attitude score	%	Univariable PRR	95% CI	*p*-value
**Age in years**	-	-	0.96	0.93–0.99	0.032
**Sex**	-	-	-	-	0.877
Female	29/47	61.7	1.00	-	-
Male	21/35	60.0	0.97	0.68–1.39	-
**In a relationship or married**	-	-	-	-	0.014
No	9/25	36.0	1.00	-	-
Yes	41/57	71.9	2.00	1.15–3.47	-
**Consultant**	-	-	-	-	0.178
No	36/54	66.7	1.08	0.73–1.61	-
Yes	14/28	50.0	1.00	-	-
**First or second year registrar**	-	-	-	-	0.702
No	14/22	63.6	1.00	-	-
Yes	22/32	68.8	1.08	0.73–1.61	-
**Qualified 2020 or later**	-	-	-	-	0.700
No	10/19	52.6	1.18	0.50–2.50	-
Yes	4/9	44.4	1.00	-	-
**Speciality**	-	-	-	-	0.272
Other	6/15	40.0	1.00	-	-
General medicine	8/13	61.5	1.54	0.71–3.32	-

PRR, prevalence rate ratio; CI, confidence interval.

Most respondents do not routinely test for pregnancy or ask women about the first date of their last normal menstrual period (LNMP) – a question that could prompt a pregnancy test if there is a history of amenorrhoea. Only 10% of those surveyed said they screen for pregnancy at every visit, and an even lower 2% will record the date of their LNMP in their notes. This was even lower than Lohr’s finding of 17% of doctors working in Internal Medicine routinely providing contraceptive counselling when seeing women of reproductive age.^12^

‘Non-pregnancy related infections’ and ‘medical disorders’ are the two highest causes contributing to the alarming maternal mortality rates in SA, reported as 109.6 per 100 000 by the NCCEMD (National Committee for the Confidential Enquiry into Maternal Deaths) in 2022.^9^ The economic inequality in the South African population is evident in the high incidence not only of communicable diseases such as HIV and tuberculosis (TB) but also of diseases of lifestyle.^13^ Hopkins et al. identified chronic hypertension, diabetes and obesity to be at their highest rate in Soweto, Gauteng, when compared with the rest of SA.^14^ Conceiving when a medical condition, such as diabetes, is uncontrolled makes the pregnancy high risk and increases the likelihood of adverse maternal and foetal outcomes.^9^ Thus, it appears that one of the most practical ways of reducing maternal morbidity and mortality may be to increase access to effective and safe contraception.^5^

The results suggest that the WHO MEC wheel (a tool for assessing and comparing various contraception methods in specific medical conditions)^15^ is not well known or commonly used by respondents, and physicians do not feel confident assessing the need for or changing patients from one method of contraception to another. One of the barriers to initiating contraception that was identified was the false idea that contraception cannot be started until the next menstrual period or unless certain investigations, such as cervical smears, are performed. This aligns with the findings of Akkers et al.^11^ and indicates that the decisions doctors make to discuss contraception with their patients are biased towards their perceived knowledge of the subject.

The knowledge score should also be interpreted in the context of the level of knowledge expected of physicians in South Africa. Although the questionnaire was not designed as a specialist examination, the Fellowship of the College of Physicians of South Africa [FCP(SA)] provides a relevant contextual benchmark, as this is the national specialist qualification for Internal Medicine physicians. The FCP(SA) written examination uses Cohen’s method of standard setting for the single-correct-answer and objective test components, where the pass mark is calculated as 65% of the 95th percentile of the scores achieved by candidates sitting the examination.^16^ Applying this benchmark illustratively to the knowledge section of our questionnaire, only 30% of respondents would have met this standard. Even when using a simple 50% threshold, only 42% of respondents would have achieved this level. This comparison should not be interpreted as a validated pass mark for this questionnaire, but it provides useful context for the finding that contraception-related knowledge among respondents was limited.

The South African population has a significant burden of non-communicable diseases, including obesity, hypertension and diabetes,^17^ all of which are known contributors to adverse maternal outcomes and maternal mortality. Comprehensive counselling and the provision of contraception are essential to the health of women of childbearing age. It is well known that pregnancy is a high-risk time in a woman’s life,^5^ and this risk is compounded when dealing with concurrent medical conditions.^2^ By delaying fertility, contraception also allows time for women who have medical conditions to achieve disease control or remission before falling pregnant.

A perceived lack of time to address family planning, with more than half of the respondents (54%, Figure 2) saying the workload in their facility is too great to provide contraceptive counselling in addition to the care they are already required to provide, was one of the main reasons for not addressing contraception with their patients. This is not unique to doctors working in Johannesburg; doctors working in hospitals in America also struggle with limited time as well as competing medical priorities.^11^

Internal Medicine doctors are uniquely positioned to assess pregnancy readiness by reaching a point where chronic medical conditions in their patients are optimised. They are managing patients when a new diagnosis is made as well as providing ongoing care for chronic medical conditions. This study draws attention to the fact that medically complex patients are more likely to have high-risk pregnancies. Moreover, the triennial report by the NCCEMD shows that unplanned pregnancies in high-risk medical groups have contributed to the high rate of maternal death in SA.^9^

Physicians in SA treat a variety of medical conditions in women; however, they are not well versed in the non-contraceptive benefits contraception use offers. Figure 3 shows the answers to the question about oral and intrauterine contraceptive methods and how they can prevent the development of endometrial cancer,^18^ the 5th most common cancer to affect women in SA after breast, cervical, colorectal and skin cancer.^19^ Patients with coexisting medical conditions often have contraindications to using oestrogen-containing contraception, and a basic knowledge of long-acting reversible contraception was lacking, despite it being the most effective form in most cases. This highlights a need for more structured training in reproductive health within Internal Medicine training programmes.

**FIGURE 3:**
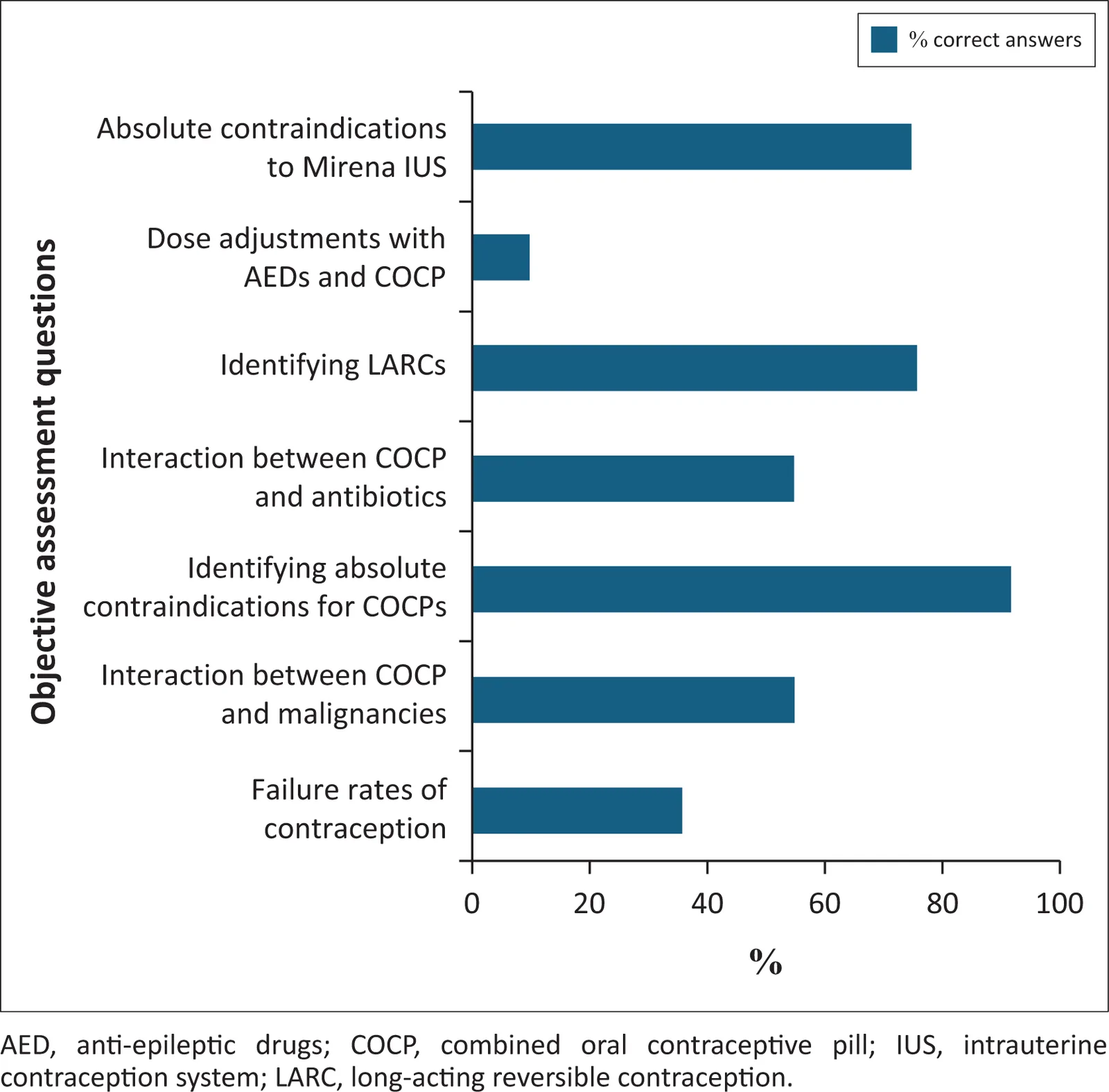
Percentage of participants who correctly answered objective assessment questions.

### Limitations

One of the limitations of this study was sampling bias: doctors who had an interest in women’s health and contraception were more likely to answer the questionnaire. In addition, because the sample size was small, the results possibly did not reflect the knowledge and practices of the whole Department of Internal Medicine. Furthermore, the study was carried out at a single university, and the Consultants were not assessed on where they did their speciality training. Finally, doctors who did not feel confident to answer questions on contraception may not have participated in the survey.

### Recommendations

The collaboration between Internal Medicine and Obstetrics and Gynaecology is crucial for the overall health of women of childbearing age, especially those with medical co-morbidities. A combined contraceptive clinic run by both departments might be able to bridge the gap in knowledge and strengthen clinical working relationships. There is a need for a family planning or pre-conception clinic to which doctors can refer patients with comorbid medical conditions for counselling and implementation of contraception. Future research could survey the population of doctors working in the private sector in South Africa and compare the answers of those physicians to those in this study.

## Conclusion

This study is important because it highlights the responsibility of Internal Medicine doctors who manage women of childbearing age, especially those women with chronic medical conditions, where pregnancy is high risk. It is vital that physicians attending to these women play a role in preventing pregnancy. Provision of a thorough care plan for such patients requires a basic understanding of the contraceptive options available, how they work and their side effects.

The findings of this study indicate that there are significant gaps in knowledge of contraception among Internal Medicine doctors. There are also poor or misinformed decision-making and counselling about contraception, which may contribute to unintended and high-risk pregnancies. These findings highlight the responsibility of doctors who manage women of childbearing age to have a basic understanding of the contraceptive options available, how they work and their side effects so that they can provide a thorough care plan for patients.

## References

[1] Yaya S, Uthman O.A., Etkoluenetale M, Bishwajit G. Women empowerment as an enabling factor of contraceptive use in sub-Sahara Africa: A multilevel analysis of cross-sectional surveys of 32 countries. Reprod Health. 2018;15(1):214. 10.1186/s12978-018-0658-5 PMC6302468 30572927

[2] World Health Organization. Maternal mortality [homepage on the Internet]. 2019 [cited 2024 Nov 10]. Available from: https://www.who.int/news-room/fact-sheets/detail/maternal-mortality

[3] Woldesenbet S, Kufa T, Lombard C. et al. The prevalence of unintended pregnancy and its association with HIV status among pregnant women in South Africa, a national antenatal survey, 2019. Sci Rep. 2021;11(1):23740. 10.1038/s41598-021-03096-z PMC8660789 34887462

[4] Gaffield M, Egan S, Temmerman M. It’s about time: WHO and partners release programming strategies for postpartum family planning. Glob Health Sci Pract. 2014;2(1):4–9. 10.9745/GHSP-D-13-00156 PMC4168603 25276558

[5] Ahmed S, Li Q, Liu L, Tsui A. Maternal deaths averted by contraceptive use: An analysis of 172 countries. Lancet. 2012;380(9837):111–125. 10.1016/S0140-6736(12)60478-4 22784531

[6] Rucinski K, Powers K, Schwartz S et al. Longitudinal patterns of unmet need for contraception among women living with HIV on antiretroviral therapy in South Africa. PLoS One. 2018;13(12):e0209114. 10.1371/journal.pone.0209114 PMC6301780 30571702

[7] Sedgh G, Ashford L, Hussain R. Unmet need for contraception in developing countries: Examining women’s reasons for not using a method [homepage on the Internet]. Guttmacher Institute; 2016 [cited 2023 Dec 15]. Available from: https://www.guttmacher.org/report/unmet-need-for-contraception-in-developing-countries

[8] United Nations Population Fund (UNFPA). State of World Population 2022: Seeing the unseen: the case for action in the neglected crisis of unintended pregnancy [Internet]. New York: UNFPA; 2022 [cited 2023 Jun 30]. Available from: https://reliefweb.int/report/world/state-world-population-2022-seeing-unseen-case-action-neglected-crisis-unintended

[9] Baloyi K, Cebekhulu S. National committee for confidential enquiry into maternal deaths: Annual report for 2023/24. [cited 2023 May 03]. Available from: https://www.health.gov.za/wp-content/uploads/2024/10/Saving-Mothers-Report-2023.pdf

[10] Nulty D. The adequacy of response rates to online and paper surveys: What can be done? Assess Eval High Educ. 2008;33(3):301–314. 10.1080/02602930701293231

[11] Akers A, Gold M, Borrero S, Santucci A, Schwarz E. Providers’ perspectives on challenges to contraceptive counseling in primary care settings. J Women’s Health. 2010;19(6):1163–1170. 10.1089/jwh.2009.1735 PMC2940510 20420508

[12] Lohr P, Schwarz E, Gladstein J, Nelson A. Provision of contraceptive counseling by internal medicine residents. J Women’s Health. 2008;18(1):127–131. 10.1089/jwh.2008.0809 19072725

[13] Dehlendorf C, Rodriguez M, Levy K, Borrero S, Steinauer J. Disparities in family planning. Am J Obstet Gynecol. 2010;202(3):214–220. 10.1016/j.ajog.2009.08.022 PMC2835625 20207237

[14] Hopkins KL, Hlongwane K, Otwombe K, et al. Demographics and health profile on precursors of non-communicable diseases in adults testing for HIV in Soweto, South Africa: A cross-sectional study. BMJ Open. 2019;9(12):e030701. 10.1136/bmjopen-2019-030701 PMC6924733 31843823

[15] World Health Organisation: Guidelines Review Committee. Medical eligibility criteria for contraceptives [homepage on the Internet]. Geneva: WHO; 2015 [cited 2024 Apr 30]. Available from: https://www.who.int/publications/i/item/9789241549158

[16] Piasta S, Justice L. Cohen’s d statistic. Encyclopedia of research design [homepage on the Internet]. 2022 [cited 2023 Mar 15]. Available from: https://sk.sagepub.com/ency/edvol/the-sage-encyclopedia-of-research-design-2e/chpt/cohen-s-d-statistic#_

[17] Haffejee F, O’Connor L, Govender N, et al. Factors associated with unintended pregnancy among women attending a public health facility in KwaZulu-Natal, South Africa. S Afr Fam Pract. 2017;60(3):79–83. 10.1080/20786190.2017.1396790

[18] Burchardt N, Shafrir A, Kaaks R, Tworoger S, Fortner R. Oral contraceptive use by formulation and endometrial cancer risk among women born in 1947–1964: The nurses’ health study II, a prospective cohort study. Eur J Epidemiol. 2020;36(8):827–839. 10.1007/s10654-020-00705-5 PMC8416825 33331993

[19] The Cancer Association of South Africa. Women & Cancer [homepage on the Internet]. CANSA – The Cancer Association of South Africa. 2024 [cited 2024 Feb 01]. Available from: https://cansa.org.za/womens-health/

